# Supplementary material for: The Role of Growth Factors in the Pathogenesis of Dengue: A Scoping Review
Source: Pathogens. 2022 Oct 13;11(10):1179. doi: 10.3390/pathogens11101179 (PMC9608673; doi:10.3390/pathogens11101179)
Supplement: Supplementary file 1 [file pathogens-11-01179-s001.zip › pathogens-1903557-supplementary.pdf]

Supplementary Table S1. Search strategy in the MEDLINE and EMBASE database (Date:04/03/2022)

MEDLINE database		
**Search**	**Query**	**Records retrieved**
#1	(Dengue[Mesh]) OR (Breakbone Fever) OR (Fever, Breakbone) OR (Classical Dengue Fever) OR (Classical Dengue Fevers) OR (Dengue Fever, Classical) OR (Break-Bone Fever) OR (Break Bone Fever) OR (Fever, Break-Bone) OR (Dengue Fever) OR (Fever, Dengue) OR (Classical Dengue) OR (Classical Dengues) OR (Dengue, Classical) OR (Severe Dengue[Mesh]) OR (Dengue, Severe) OR (Dengues, Severe) OR (Severe Dengues) OR (Dengue Hemorrhagic Fever) OR (Thai Hemorrhagic Fever) OR (Fever, Thai Hemorrhagic) OR (Philippine Hemorrhagic Fever) OR (Fever, Philippine Hemorrhagic) OR (Singapore Hemorrhagic Fever) OR (Fever, Singapore Hemorrhagic) OR (Hemorrhagic Fever, Dengue) OR (Hemorrhagic Dengue) OR (Hemorrhagic Dengues) OR (Dengue Shock Syndrome)	26,834
#2	(Chikungunya Fever[Mesh]) OR (Chikungunya Fevers) OR (Fever, Chikungunya) OR (Chikungunya Virus Infection) OR (Chikungunya Virus Infections) OR (Infection, Chikungunya Virus) OR (Chikungunya Fever) OR (Chikungunya Fevers) OR (Fever, Chikungunya)	5,485
#3	(Zika Virus Infection[Mesh]) OR (Infection, Zika Virus) OR (Virus Infection, Zika) OR (ZikV Infection) OR (Infection, ZikV) OR (Fever, Zika) OR (Zika Virus Disease) OR (Disease, Zika Virus) OR (Virus Disease, Zika) OR (Zika Fever) OR (Congenital Zika Syndrome) OR (Congenital Zika Virus Infection)	8,817
#4	(Platelet-Derived Growth Factor[Mesh]) OR (Factor, Platelet-Derived Growth) OR (Growth Factor, Platelet-Derived) OR (Platelet Derived Growth Factor) OR (PDGF)	32,459
#5	(Epidermal Growth Factor[Mesh]) OR (Growth Factor, Epidermal) OR (Urogastrone) OR (EGF) OR (Human Urinary Gastric Inhibitor) OR (beta-Urogastrone) OR (beta Urogastrone) OR (Epidermal Growth Factor-Urogastrone) OR (Growth Factor-Urogastrone, Epidermal)	95,013
#6	(Hepatocyte Growth Factor[Mesh]) OR (Factor, Hepatocyte Growth) OR (Growth Factor, Hepatocyte) OR (Scatter Factor) OR (Factor, Scatter) OR (Hepatopoietin) OR (Hepatopoietin A)	17,842
#7	(Vascular Endothelial Growth Factors[Mesh]) OR (VEGF)	91,105
#8	(Fibroblast Growth Factor[Mesh]) OR (Growth Factors, Fibroblast) OR (Fibroblast Growth Factor) OR (Growth Factor, Fibroblast) OR (Fibroblast Growth Regulatory Factor)	64,803
#9	(Granulocyte Colony-Stimulating Factor[Mesh]) OR (G-CSF)	45,593
#10	(Granulocyte-Macrophage Colony-Stimulating Factor[Mesh]) OR (Granulocyte Macrophage Colony Stimulating Factor) OR (CSF-2) OR (Tumor-Cell Human GM Colony-Stimulating Factor) OR (Tumor Cell Human GM Colony Stimulating Factor) OR (GM-CSF) OR (Histamine-Producing Cell-Stimulating Factor) OR (Cell-Stimulating Factor, Histamine-Producing) OR (Histamine Producing Cell Stimulating Factor) OR (TC-GM-CSF) OR (Colony-Stimulating Factor, Granulocyte-Macrophage) OR	32,566

	(Colony Stimulating Factor, Granulocyte Macrophage) OR (CSF-GM)	
#11	(Transforming Growth Factors[Mesh]) OR (Factors, Transforming Growth) OR (Growth Factors, Transforming) OR (Transforming Growth Factor) OR (Factor, Transforming Growth) OR (Growth Factor, Transforming) OR (TGF)	125,442
#12	(Insulin-Like Growth Factor I[Mesh]) OR (Insulin-Like Somatomedin Peptide I) OR (Insulin Like Somatomedin Peptide I) OR (Somatomedin C) OR (IGF-I-SmC) OR (IGF-1) OR (IGF-I) OR (Insulin Like Growth Factor I)	51,677
#15	#1 OR #2 OR #3	35,617
#16	#4 OR #5 OR #6 OR #7 OR #8 OR #9 OR #10 OR #11 OR #12	456,181
#17	#15 AND #16	**178**
**EMBASE database**		
**Search**	**Query**	**Records retrieved**
#1	dengue OR chikungunya OR 'Zika fever'	47,861
#2	'epidermal growth factor'	218,307
#3	'platelet derived growth factor'	54,833
#4	'scatter factor'	28,552
#5	'vasculotropin'	174,762
#6	'fibroblast growth factor'	95,695
#7	'granulocyte colony stimulating factor'	62,208
#8	'granulocyte macrophage colony stimulating factor'	50,551
#9	'transforming growth factor'	182,455
#10	'somatomedin'	103,323
#11	#2 OR #3 OR #4 OR #5 OR #6 OR #7 OR #8 OR #9 OR #10	806,951
#12	#1 AND #11	**556**
